# Mass Sensors Based on Capacitive and Piezoelectric Micromachined Ultrasonic Transducers—CMUT and PMUT

**DOI:** 10.3390/s20072010

**Published:** 2020-04-03

**Authors:** Haleh Nazemi, Jenitha Antony Balasingam, Siddharth Swaminathan, Kenson Ambrose, Muhammad Umair Nathani, Tara Ahmadi, Yameema Babu Lopez, Arezoo Emadi

**Affiliations:** Department of Electrical and Computer Engineering, University of Windsor, Windsor, ON N9B 3P4, Canada; nazemih@uwindsor.ca (H.N.); antonyb@uwindsor.ca (J.A.B.); swaminas@uwindsor.ca (S.S.); kenson.ambrose@uwindsor.ca (K.A.); nathan11@uwindsor.ca (M.U.N.); ahmadit@uwindsor.ca (T.A.); babulop@uwindsor.ca (Y.B.L.)

**Keywords:** capacitive micromachined ultrasonic transducer (CMUT), gas detection, low concentration, mass sensors, microelectromechanical systems (MEMS), microfabrication, piezoelectric micromachined ultrasonic transducer (PMUT), volatile organic compounds (VOC)

## Abstract

Microelectromechanical system (MEMS)-based mass sensors are proposed as potential candidates for highly sensitive chemical and gas detection applications owing to their miniaturized structure, low power consumption, and ease of integration with readout circuits. This paper presents a new approach in developing micromachined mass sensors based on capacitive and piezoelectric transducer configurations for use in low concentration level gas detection in a complex environment. These micromachined sensors operate based on a shift in their center resonant frequencies. This shift is caused by a change in the sensor’s effective mass when exposed to the target gas molecules, which is then correlated to the gas concentration level. In this work, capacitive and piezoelectric-based micromachined sensors are investigated and their principle of operation, device structures and configurations, critical design parameters and their candidate fabrication techniques are discussed in detail.

## 1. Introduction

Microelectromechanical system (MEMS)-based sensors are introduced as high-performance detectors due to their sensing capabilities at the micro and nanoscale levels and their potential for integration with wearable electronics [1,2]. These gas sensors can employ various detection techniques using resistivity, optical properties, acoustic measurements and mass detection [3]. Amongst them, the mass detection method is reported as an emerging candidate due to its stellar performance in detecting low gas concentration levels [3]. MEMS-based mass resonant sensors benefit from a low power consumption, a high selectivity and a low limit of detection (LOD) whilst being integrable in a wide range of applications [1]. Amongst them, micromachined ultrasonic transducers (MUT) are shown as potential candidates in sensing applications [4,5,6]. In an unconventional approach, these devices can be employed in gas sensing technology due to their unique structures that provide design flexibility and contribute to their potential high-performance capabilities. MUT-sensors operate based on a change in the mass of their sensing components when used for gas sensing and volatile organic compound (VOC) detection. In addition, MUT devices benefit from advanced micro fabrication technologies, and therefore, these micromachined-sensors can be fabricated in an array structure using various sensing materials in order to improve their selectivity in complex environments [4,5,7]. A common attribute of the mass loading effect is the implementation of the proposed capacitive and piezoelectric micromachined ultrasonic transducers, capacitive micromachined ultrasonic transducer (CMUT) and piezoelectric micromachined ultrasonic transducer (PMUT) structures, that are dynamically driven into mechanical deformation. This deflection creates a detectable shift in the frequency of their resonating plane due to the adsorption of the gas molecules on the sensing membrane, usually a polymer which is chosen based on the environment and analyte detection requirements [4]. In these sensors, adsorption interactions between the gas molecules and the polymer occurs via physisorption that influences sensor response time during gas adsorption and desorption. In this work, CMUT and PMUT-based sensors alongside their structural mechanisms, device structures and implementation methods based on mass shift are discussed in detail.

## 2. Capacitive Micromachined Ultrasonic Transducer-Based Gas Sensor

### 2.1. Introduction

CMUTs were introduced as an alternative device for the traditional piezoelectric transducers for non-destructive testing applications as well as medical and underwater imaging [8,9]. Due to the unique structure of the CMUTs, they can also act as a mass resonant sensor [10]. Therefore, in an unconventional approach, they are proposed to be used for volatile organic compound detection applications. A CMUT benefits from a simple parallel plate structure and, as a gas sensor, it can be functionalized by depositing a polymer sensing layer. The designed sensing material absorbs the gas molecules and hence, the sensor reacts to a change in the sensing layer mass when exposed to target gas molecules.

### 2.2. CMUT Sensor Structure and Mechanism of Operation

The structure of a CMUT includes a deflectable top membrane that is suspended over a fixed bottom electrode [11]. The top membrane is clamped at the edges. The device’s top membrane is commonly metalized or is fabricated using a highly conductive material [12,13].

(1) $$k=\frac{16\;\pi \;E_{m}\;t_{m}^{3}}{3\;\left(1-\upsilon_{m}^{2}\right)\;r_{m}^{2}}-\frac{\varepsilon_{0}\;A_{m}\;V^{2}\;}{h_{eff}^{3}}+4\pi \sigma_{m}t_{m}$$

In Equation (1), the terms *E_m_*, *t_m_*, *r_m_*, *υ_m_*, *A_m_*, *σ_m_*, *V* and *h_eff_* are the membrane’s Young’s modulus, thickness, radius, Poisson’s ratio, area, residual stress, the applied DC voltage and the device’s effective cavity height, respectively. In Equation (1), the first term is defined by the membrane’s geometry and its material properties, while the second and third terms are known as the spring softening effect due to the applied DC bias voltage and the membrane’s residual stress, respectively [13].

(2) $$V_{pull-in}=\sqrt{\frac{8kh_{0}^{3}}{27\varepsilon_{0}A_{m}}}$$

**Figure 1:**
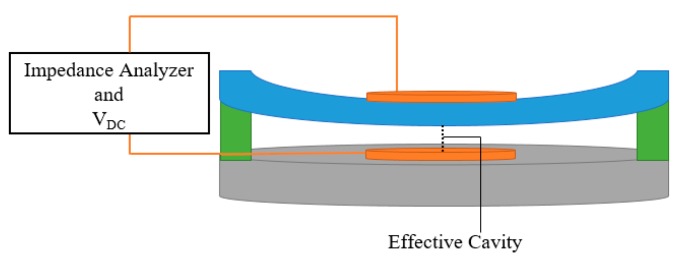
Schematic view of a capacitive micromachined ultrasonic transducer (CMUT) with deflected top membrane.

In Equation (2), *h_0_* and *ε_0_* are the initial and unbiased cavity height, and the permittivity of the vacuum, respectively. By employing the spring softening constant in the Equation (3),

(3) $$\omega_{r}=2\pi \;f_{r}=\sqrt{k/m_{m}}$$

where m_m_ is the effective mass of the membrane, the central resonant frequency of the device is approximated [13]. In order to measure the resonant frequency, the device top membrane and bottom electrode are connected to an impedance analyzer, as shown in Figure 1.

**Figure 2:**
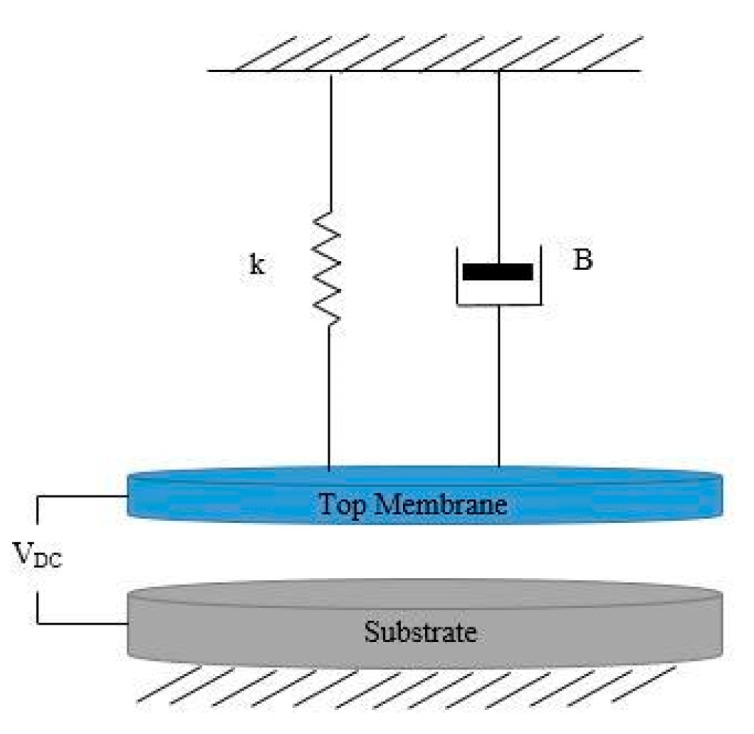
Schematic view of a mass-spring-damper model of a CMUT-based gas sensor.

The CMUT sensor is modeled using a mass-spring-damper in one dimension, as shown in Figure 2, where *k* and *B* are the membrane’s spring constant and damping factor, respectively [10]. In the proposed analytical models of the CMUT, two different approaches exist which address the device membranes’ small and large deflections. The small deflection approach is proposed when the membrane’s displacement due to the sensing material’s mass change is small in comparison to its thickness, and there is a linear relation between the applied force and the membrane’s displacement [10]. Meanwhile, in the large membrane deflections, there is a nonlinearity seen between the membrane’s displacement and the applied force [14]. 

**Figure 3:**
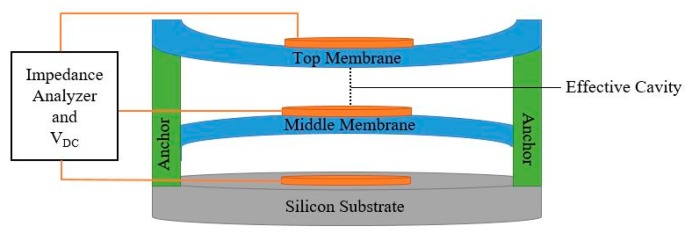
Schematic view of an M^3^-CMUT.

CMUT sensors can also be developed with a multiple moving membrane capacitive micromachined ultrasonic transducer (M^3^-CMUT) configuration [15]. This configuration, shown in Figure 3, benefits from two or more deflectable membranes that contribute to the device’s performance.

**Figure 4:**
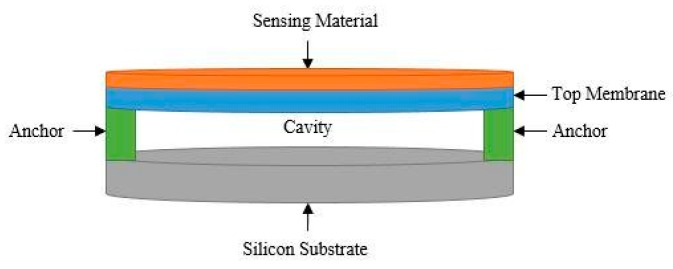
Schematic view of a CMUT-based gas sensor with a deposited sensing material.

In this design, a DC bias voltage is applied either to the top or the middle membranes, while the bottom electrode is grounded and shorted to the unbiased flexible membrane. This biasing configuration introduces an attraction between the membranes. Consequently, a smaller cavity height can be achieved at a lower DC bias voltage. This, in return, results in enhanced operational properties, and higher sensitivity [15]. When used as a gas sensor, the top membrane is functionalized by a sensing material, as shown in Figure 4. Various polymers can be used as sensing materials in gas sensing applications including polyisobutylene (PIB) [16] and polydimethylsiloxane (DKAP) [17]. When a CMUT gas sensor is exposed to a target gas, the sensing material absorbs the target gas molecules, which, in turn, causes a change in the sensing material’s effective mass and thickness, hence prompting changes in the top membrane’s effective mass. This change in the device’s effective mass and sensing component’s thickness creates a shift in the sensor’s central resonant frequency, as shown in Equation (3), which can be correlated to the concentration level of the target gas molecules.

**Figure 5:**
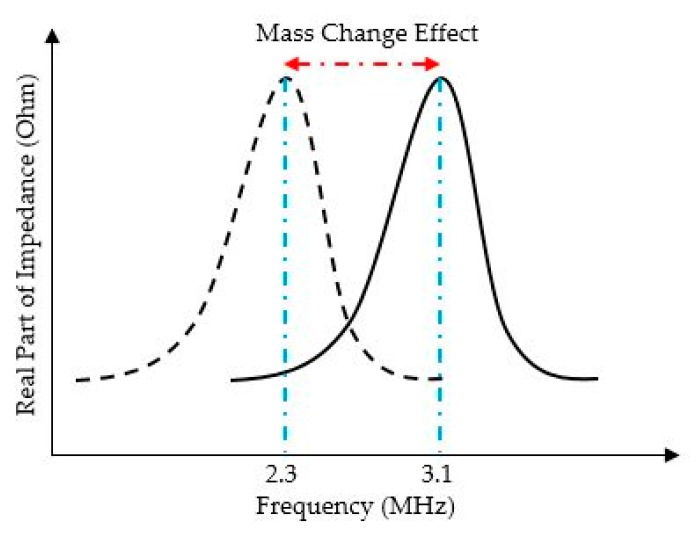
Frequency shift in response to the mass change for a CMUT sensor with top membrane radius of 25 μm, thickness and cavity height of 500 nm, functionalized by a 300 nm PIB operating.

The device sensitivity is defined by the sensor physical and material properties presented in Equations (1) to (3). Moreover, the sensitivity is influenced by the designed sensing material [10,18]. A frequency shift, from 3.1 to 2.3 MHz, for a simulated CMUT-based sensor was achieved when the sensor absorbed 1.7 fg of the target gas molecules, as illustrated in Figure 5. This device operates with a 30 V DC bias applied to the top membrane. This sensor is functionalized by a 300 nm PIB sensing layer. The top membrane’s radius is 25 μm and its thickness and cavity height are 500 nm. 

**Figure 6:**
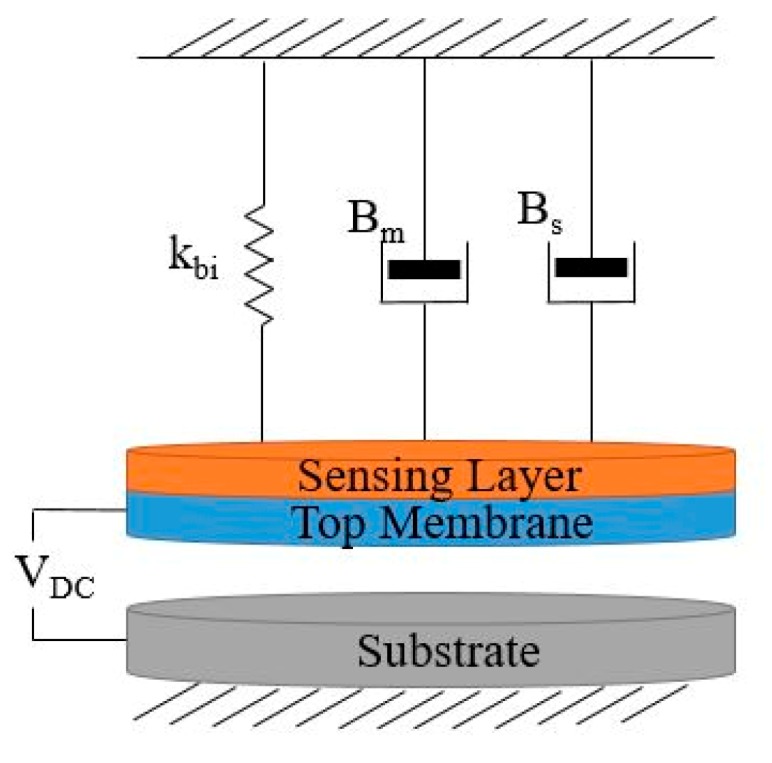
Schematic view of a mass-spring-damper model of CMUT-based gas sensor.

In a CMUT-based sensor, the sensing component and the absorbed mass of gas act together as an added mass to the membrane and spring, as shown in Figure 6. Therefore, a comprehensive model is proposed that further includes the damping effect, *B_s_*, of this added mass, in addition to the membrane’s damping, *B_m_* [18]. 

This added mass affects the spring constant, the sensing material properties, and the sensing layer’s dimensions and the membrane’s stress, which, consequently, affect the spring force by changing the spring constant to *k_bi_*, as shown in Equation (4),

(4) $$F=-k_{bi}x$$

where *k_bi_* and *x* are the deflectable membrane’s stiffness and the displacement, respectively. Moreover, the added mass of the sensing material and the absorbed gas molecules to the mass of membrane alters the resonant frequency of the sensor from Equation (3) to Equation (5) [18],

(5) $$\omega_{r}=2\pi \;f_{r}=\sqrt{\frac{k_{bi}}{m_{m}+m_{s}+∆m}}$$

where *m_m_*, *m_s_* and *∆m* are masses of the membrane, the sensing material and the absorbed gas, respectively. Therefore, in order to design a CMUT-based sensor for a target gas detection application, effective structural parameters should be considered in addition to the sensing component’s properties to enhance device sensitivity. These critical parameters consist of the membrane’s radius and thickness, the cavity height and the structural material in addition to the sensing component’s thickness and its material properties [10,18]. 

An analytical model is accordingly developed for bi-layer circular CMUT-based gas sensors that includes the sensor’s physical parameters, the material properties of the structural and sensing layers, the residual stresses of the sensing layer, and the thermal and intrinsic stresses of the top membrane, as well as the membrane’s stiffness and softening effect due to the applied DC bias voltage, as shown in Equation (6) [10],

(6) $$k_{bi}=\frac{64\;\pi \;D_{eff}}{\;r_{m}^{2}}-\frac{\varepsilon_{0}\;A_{m}\;V^{2}\;}{h_{eff}^{3}}+4\pi (\sigma_{m}t_{m}+\sigma_{s}t_{s})$$

where *σ_s_* and *t_s_* represent the sensing material’s residual stress and its thickness, respectively. *D_eff_* is the effective flexural rigidity and it is defined in Equation (7) and derived by employing Equation (8) through Equation (10) in Equation (7).

(7) $$D_{eff}=\frac{AC-N^{2}}{A}$$

(8) $$A=\frac{E_{m}}{1-\upsilon_{m}^{2}}t_{m}+\frac{E_{s}}{1-\upsilon_{s}^{2}}t_{s}$$

(9) $$N=\frac{E_{m}}{2\;\left(1-\upsilon_{m\;}^{2}\right)}t_{m}^{2}+\frac{E_{s}}{2\;\left(1-\upsilon_{s}^{2}\right)}\left({\left(t_{s}+t_{m}\right)}^{2}-t_{m}^{2}\right)$$

(10) $$C=\frac{E_{m}}{3\;\left(1-\upsilon_{m\;}^{2}\right)}t_{m}^{3}+\frac{E_{s}}{3\;\left(1-\upsilon_{s}^{2}\right)}\left({\left(t_{s}+t_{m}\right)}^{3}-t_{m}^{3}\right)$$

In this model, the sensitivity, defined as the frequency shift per unit mass change, as seen in Equation (11), can reach the Hz/zg level [10]. A sensitivity of 364 Hz/zg is achieved for a CMUT-based gas sensor with a 7 μm radius and a 500 nm polysilicon membrane thickness functionalized by a 300 nm PIB layer [10].

(11) $$S=\frac{∆f}{∆m}$$

Furthermore, mass sensitivity per unit area is defined by Equation (12), which indicates that smaller radii and lower density provide a higher mass sensitivity in CMUT-based gas sensors [17,18]. The recently reported mass sensitivity per unit area is 130 zg/Hz/μm^2^ for structures with 9 μm radii and 500 nm membrane thicknesses [11], which is further improved to 48.8 zg/Hz/μm^2^ for devices with 5.3 μm structural radii and 500 nm membrane thicknesses [12]. The aforementioned sensors are used for dimethyl methylphosphonate (DMMP) detection.

(12) $$S_{m}=-2\;\frac{m}{f_{0}A_{m}}=-2\frac{\rho t}{f_{0}}$$

The device illustrated in Figure 4 with the presented frequency shift in Figure 5 provides a sensitivity of 30 Hz/zg. 

### 2.3. CMUT Sensor Microfabrication Techniques

Advanced micromachining techniques are used to fabricate CMUT-based gas sensors. Employing these technologies provides batch fabrication, high yield and uniformity, ability for array fabrication, and an ease of circuit integration due to the material properties of silicon as the substrate [11,16]. The two most common CMUT fabrication techniques are the sacrificial release process [19], and the wafer bonding process [4], both of which are investigated and discussed in this paper. Utilizing advanced microfabrication technology, individual CMUT cells can be fabricated to form an array of sensors with various arrangements including circular, square, O-ring, as well as hexagonal shapes [20,21,22]. This ability to form an array of sensors on the same chip is beneficial when a CMUT is designed to detect a target gas in a complex environment. Employing an array configuration can address the disadvantages associated with the lack of selectivity for the commonly used polymers as the sensing layers [11].

#### 2.3.1. Sacrificial Technique

**Figure 7:**
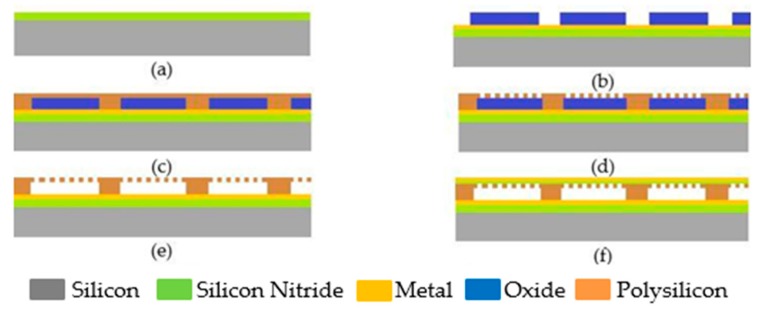
Schematic view of CMUT fabrication steps using sacrificial release process. (**a**) depositing silicon nitride as the insulator and etch-stop-layer, (**b**) depositing bottom electrode and silicon dioxide followed by patterning silicon dioxide as the sacrificial layer, (**c**) depositing silicon nitride or polysilicon as the top membrane, (**d**) Patterning releasing holes on the top membrane, (**e**) wet etching silicon dioxide to release the top membrane, (**f**) depositing top electrode on the membrane.

The sacrificial fabrication process is a standard fabrication technique used for CMUTs. In this technique, a highly doped silicon substrate is covered by a thin insulator layer such as silicon nitride, which acts as the etch-stop-layer for the next fabrication step, as illustrated in Figure 7a [23]. In the next step, a layer of metal is deposited as the bottom electrode, which is followed by depositing and patterning silicon dioxide, SiO_2_, as the sacrificial layer (Figure 7b). To create the top membrane, a layer of polysilicon or silicon nitride is deposited on the sacrificial layer (Figure 7c). In order to release the top membrane and form the gap, etch holes are created on the top membrane to reach the underneath layer. After removing the sacrificial layer through a wet etching process in Figure 7d and e, a cavity is formed between the silicon substrate and the membrane. The device is then vacuum sealed by depositing another silicon nitride layer on top of the structure [23,24]. In the next step, a metal layer is deposited on the top membrane to create the top electrode, as shown in Figure 7f. To functionalize the device as a gas sensor, a sensing material is deposited on the top membrane. In order to deposit the sensing material, different techniques can be used, including inkjet printing, spray coating, drop casting and polymer evaporation. The sacrificial technique is reported to be used for CMUT devices with membrane radii ranging over tens of microns [23].

#### 2.3.2. Wafer Bonding Techniques

**Figure 8:**
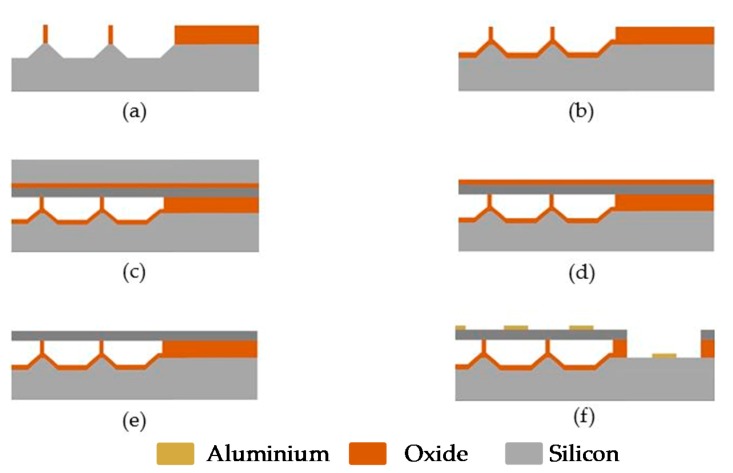
Schematic view of CMUT fabrication steps using wafer fusion bonding technique. (**a**) thermally growing silicon dioxide followed by patterning it to create the cavity, (**b**) growing silicon dioxide to insulate the substrate followed by oxide buffering and wafer cleaning process, (**c**) bonding SOI wafer and the first substrate followed by annealing them to strengthen Van der Waals bonds, (**d**) grinding and wet etching SOI handle wafer, (**e**) wet etching BOX layer to release the top membrane, (**f**) sputtering and patterning metal electrode on the top membrane.

Although the sacrificial release process has been widely used for CMUT fabrication due to its low cost and ease of fabrication, the wafer bonding technique provides a better control over the membrane thickness and cavity height in addition to a lower residual stress during fabrication. One of the constraints of the wafer bonding process is the membrane’s surface roughness and cleanliness before bonding [25]. In the wafer bonding fabrication process, two separate substrates are used. One highly doped silicon substrate is used to create the bottom electrode and the cavity, while the other employs a silicon on insulator (SOI) wafer that is used for the top membrane. Wafer fusion bonding for CMUT-based sensor fabrication is reported for devices with radii ranging from tens to hundreds of microns [26,27]. A layer of silicon dioxide is thermally grown on the first silicon substrate based on the desired cavity height which then is patterned photolithographically, as illustrated in Figure 8a. In order to create a thin insulating layer at the bottom of the cavities, another layer of silicon dioxide is thermally grown, as shown in Figure 8b. A critical point in direct wafer bonding is having smooth surfaces to create Van der Waals bonds. Therefore, wafer cleaning process is done on the SOI wafer in addition to buffering the oxide anchors on the first substrate before starting the bonding process. During the next step, both the SOI wafer and oxide surfaces on the first substrate are brought together to build Van der Waals bonds in a hydrogen chamber, which is followed by annealing at 1100 °C, as presented in Figure 8c. In order to release the top membrane, the SOI handle wafer is removed, followed by the removal of the buried oxide layer (BOX). To remove the SOI handle, the BOX layer acts as the etch stop layer while the silicon membrane plays the same role while removing the BOX layer. The main portion of the SOI handle wafer is removed by mechanical grinding, which is further followed by wet etching using potassium hydroxide (KOH) to remove the rest of it as shown in Figure 8d. The BOX layer is then removed by wet etching using buffered oxide etchant (BOE) (Figure 8e). After releasing the structure, a metal layer is deposited using the sputtering technique and patterned on the top electrode as illustrated in Figure 8f. 

A structure’s effective mass is critical in the sensitivity of the CMUT gas sensor [11]. Therefore, fabrication techniques which provide more controllable processes for a very thin top membrane are suggested to improve device sensitivity. A higher efficiency has been reported for CMUT gas sensors fabricated by the wafer bonding technique than the ones fabricated by the sacrificial technique. This is due to the optimization feasibility in the wafer bonding technique [28]. Furthermore, as seen in Equation (12), lighter membranes contribute to a higher sensitivity, which is more feasible to achieve in the wafer bonding technique. 

#### 2.3.3. Flexible CMUT Structures

Flexible CMUTs are fabricated using polymers. In order to fabricate a flexible CMUT, the sacrificial release process similar to the steps described in Section 2.3.1 can be used on a coated silicon substrate with polyethylene terephthalate (PET). SU-8 is employed as the structural material. Another layer of SU-8 on a PET substrate is roll laminated to the first wafer in order to form the top membrane. The PET substrate is subsequently removed, and platinum and gold are then deposited to form the electrode. Then, the silicon wafer is removed to provide a flexible CMUT structure [29]. This technique is used to fabricate CMUT structures with membrane radii ranging over tens of microns.

#### 2.3.4. CMUT Functionalization with Sensing Material and Device Sensitivity

As selectivity is a critical component for sensory performances, the sensing material cross-sensitivity can be addressed by using advanced microfabrication technology, wherein, individual CMUT cells can be configured in an array format [30]. In order to use CMUT configuration as a gas sensor, the top membrane can be coated by a polymer using different techniques including spin coating, inkjet dispensing, dip coating, inkjet printing, layer-by-layer deposition and electrodeposition [16,31,32,33,34,35]. 

In the spin coating technique, the polymer is diluted into a proper solvent, then it is spin coated within a specific time to achieve a uniform and desirable polymer thickness. This technique is used in functionalizing the CMUT structure with different polymers such as methylated (polyethylene-imine) (mPEI), which acts as a detector of CO_2_ and SO_2_ [4]. 

The inkjet dispensing system is also one of the technologies that is used to functionalize the CMUT structure by PIB, to detect DMMP [11]. In this technique, PIB is diluted in toluene and a droplet is dispensed on the CMUT’s top membrane. Accurately measuring the polymer thickness is not possible by optical measurement due to polymer transparency; therefore, an atomic force microscope (AFM) can be used to measure the coated polymer thickness on the CMUT structure [11]. 

Dip coating is another technique wherein the device is dipped into the functionalized material, which is diluted in the proper solvent before being taken out at a constant speed. The speed defines the thickness of the coated material [36]. The inkjet printing technique is recommended for two-dimensional individual MEMS devices, as well as for arrays. In this technique, the polymer is ejected by a deflected piezoelectric diaphragm using an applied pulsed voltage. The number of ejected droplets in this method is electrically controllable [37]. 

Surface functionalization using polymers produces gas-sensitive films which alter the resonant frequency of the device due to their imposed mass on the device when applied to the surface of a sensor [4]. Further mass changes are expected during sensing upon interaction of the analytes with the polymer, which ultimately leads to adsorption [17]. Analytes in the gas phase, such as carbon dioxide, sulfur dioxide, volatile organic compounds (VOCs) including acetone, methanol, ethanol, toluene, benzyl methyl ketone (BMK) and DMMP, and water, are viable for sensing based on mass changes of polymer films [4,27,38,39]. These gas-sensitive films comprising polymers such as poly(styrene-co-allyl alcohol), poly(vinylacetate), poly(capralactone), methylated poly(ethylene imine), poly (vinyl pyrrolidine) and poly {methyl [4- (2- hydroxy-4, 6-bistrifluoromethyl) phenyl] propylsiloxane} have been reported for detecting the analytes mentioned above [4,17,34,39,40]. Polymer properties such as specific chemical functional groups promote temporary weak interactions between analyte molecules and the polymer thus favoring physisorption. It has been shown that CO_2_ physisorbs onto methylated poly(ethylene imine) via weak molecular coordination whereas SO_2_ forms stronger irreversible hydrogen bonds with the amine group, which in turn degrades the polymer film [34]. 

VOCs such as toluene, acetone, ethanol and methanol can be detected using a CMUT-based resonant sensor array using polymers such as poly(styrene-co-allyl alcohol), poly(ethylene oxide) and poly(isobutylene) as gas-sensitive coatings [30,39]. In the case of toluene detection, the CMUT sensors had a mass sensitivity of 0.8 Hz/fg and a chemical sensitivity of 1.5 Hz/ppm. These sensitivities translate to a mass change of 4.4 pg upon exposure to the highest explored toluene concentration during sensing [30]. CO_2_ and SO_2_ can be sensed using a 16 MHz CMUT sensor functionalized using spin coating with methylated poly(ethylene imine) [38]. Once functionalized, the CMUT sensor demonstrated a 4 MHz decrease in resonant frequency due to the added mass of the gas-sensitive coating. The sensitivities for CO_2_ and SO_2_ were observed to be 8 and 20 Hz/ppm, respectively. These values represent higher sensitivities compared to other reported CMUT sensors [41]. The increased sensitivity to SO_2_ likely results from its ability to form a stronger and irreversible hydrogen bond-type interaction with the polymer during sensing. With a theoretical mass sensitivity of 0.536 Hz/fg, CO_2_ exposure induced an additive mass change of 186 fg onto the sensor during testing. A non-polymer-containing device was also tested where a Reichardt’s dye applied by drop casting was used as a coating for sensing BMK and water [27]. It was indicated that BMK adsorbs onto the dye via weak interactions such as Van der Waals and π-π interactions. Mass sensitivity was measured to be 0.24 Hz/ag, which is higher than the previously mentioned reports, wherein, added mass due to BMK adsorption was approximately 0.75 pg during sensing. Ultimately, the mass change, ∆*m*, induced by adsorption of the analyte molecules during sensing can also be estimated using equations where the other parameters are determined from experiments and sensor specifications from fabrication.

## 3. Piezoelectric Micromachined Ultrasonic Transducers-Based Gas Sensor

### 3.1. Introduction

The application of mechanical force on certain crystals and ceramics, which has no center of inversion symmetry, generates an electric charge. This phenomenon is known as the direct piezoelectric effect. The deformation of the crystal on an applied electric field generates ultrasound, is known as the indirect piezoelectric effect. This advantageous effect can be utilized to design a PMUT sensor. In convention, the inverse effect is used to construct an ultrasound transducer [42]. However, PMUT as a gas sensor relies on frequency shift as an output, approached unconventionally by utilizing the direct piezoelectric effect. A PMUT-based gas sensor can be achieved by adding a sensing layer on top of the structure of PMUT. Similar to CMUT, the material of the sensing layer plays a predominant role in determining the resonant frequency and sensitivity of the micromachined gas sensor. Several sensing methods exist, including techniques based on optical, electrical and mechanical properties. In the CMUT, as described in Section 2, a high mass sensitivity and sensing performance can be achieved. However, it also presents issues, such as the requirement of a high bias voltage and the limitation imposed by the cavity structure. PMUT gas sensors can potentially address some of the limitations. The ability to operate at lower voltages, flexibility in adapting to different sensing materials, and uncomplicated array configuration promises numerous applications in the fields of medicine, environmental monitoring and agriculture [43].

### 3.2. PMUT Sensor Structural Mechanism of Operation

**Figure 9:**
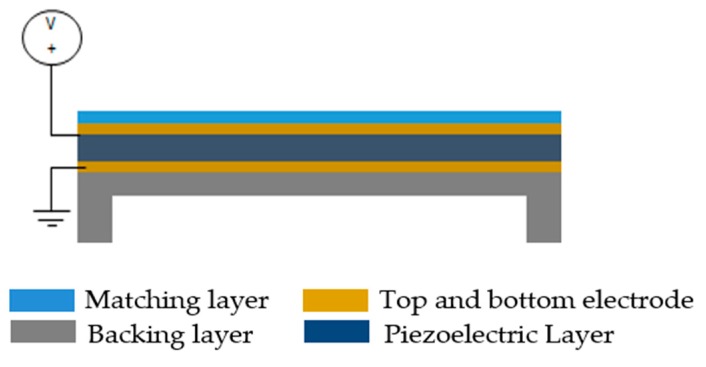
Schematic view of a piezoelectric micromachined ultrasonic transducer (PMUT).

The structure of a PMUT includes a deflectable piezoelectric membrane sandwiched between the top and bottom electrodes, as shown in Figure 9. The entire structure is clamped at the edges. When an AC voltage is applied at the top electrode, the piezo-layer vibrates at its resonant frequency due to the piezoelectric effect [44].

The piezoelectric layer deforms when an AC signal is applied to it. This is explained by the relationship between the charge co-efficient (*d*) and the voltage co-efficient (*g*) [43,44]. The applied field determines the charge generation in the charge co-efficient whereas the voltage coefficient is dependent on the charge co-efficient, the relative permittivity and the permittivity of free space.

(13) $$g=\frac{d}{\varepsilon_{0}\varepsilon_{r}}$$

(14) $$d\;=\;\frac{strain\;developed}{applied\;electric\;field}(m/v)\;=\;\;\frac{charge\;density}{applied\;mechnical\;stress}(C/N)$$

(15) $$g\;=\;\frac{open\;circuit\;electric\;field}{applied\;mechanical\;stress}(V.m/N)\;=\;\frac{strain\;developed}{applied\;charge\;density}(m/C)$$

The voltage generated in an area of *A*, thickness of *t* and relative permittivity of $\;\varepsilon_{r}$ is given by Equation (16). The subscript 3 represents the direction of polarization [43].

(16) $$V_{3}=\frac{Q_{3}}{C}=\frac{d_{33}F_{3}t}{\varepsilon_{0}\varepsilon_{r}A}$$

The strain produced by AC signals at a given frequency generates structural vibrations in the piezoelectric material layer. Unlike CMUTs, PMUTs have no vacuum gap between the top and bottom electrodes for vibration. Thus, the displacement of the membrane is not limited by the separation of the top and bottom electrodes. From the constitutive equations, the piezoelectric constants of $e_{31,f}$ and $d_{33,f}\;$ are expressed by Equations (17) and (18) [45].

(17) $$e_{31,f}=\frac{d_{31}}{s_{11}^{E}+s_{12}^{E}}\;\equiv \;e_{31}-\;\frac{c_{13}^{E}}{c_{33}^{E}}e_{33}\;|e_{31,f}|\;>\;|e_{31,f}|$$

(18) $$d_{33,f}=\frac{e_{33}}{c_{33}^{E}}\;\;\equiv \;\;d_{33}-\;\frac{2x^{E}{}_{31}}{s_{11}^{E}+s_{12}^{E}}\;\;\;\;\;\left|d_{31}\right|\;<\;|d_{33}|$$

where *e* is the piezoelectric constant, *c* is the stiffness of the material, and the subscript *f* represents the thin film. From Equation (17) and Equation (18), we see that |$e_{31,f}|$ > |$e_{31}|$ and that $\left|d_{33,f}\right|$ < $\left|d_{33}\right|$ due to higher piezoelectric constants. Also, using constitutive equations from Equations (15) and (16), the displacement field in perpendicular direction and stresses in the planar direction can be derived for the sensor and the actuator in *d_33_* mode. The displacement field in perpendicular direction is given by in-plane strain and out of plane stress, as shown in Equation (19).

(19) $$D_{3\;}=\varepsilon_{0}\varepsilon_{33,f}+e_{31,f}.\left(S_{1}+S_{2}\right)+d_{33,f}.T^{3}$$

where the dielectric constant of the film under in-plane stress is given by Equation (20).

(20) $$\varepsilon_{33,f}=\varepsilon_{33}^{T}-\frac{2d_{31}^{2}}{\varepsilon_{0}.\left(s_{11}^{E}+s_{12}^{E}\right)}$$

For a multilayer structure with a combination of thick and thin layers, the neutral plane is given by Equation (21), where *N* is the combination of different materials with differences in the Young’s modulus.

(21) $$Z_{n}=\frac{\sum_{i=1}^{N}[E_{i}\int_{h_{i-1}}^{h_{i}}\left(zdz\right)]}{\sum_{i=1}^{N_{1}}\left(E_{i}t_{i}\right)}$$

For a given material, the flexural rigidity D is a measure of a material’s resistance to bending, which is given by Equation (22).

(22) $$D_{m}=\sum_{i=1}^{N_{i}}[E_{i}\int_{h_{i-1}}^{h_{i}}{\left(z-z_{n}\right)}^{2}\mathrm{dz}]$$

For the thin film piezoelectric device, the stresses in the third axis are negotiable [45]. Therefore, the sensitivity, *G_s_*, of a thin film piezoelectric device is given by Equation (23).

(23) $$G_{s}\;\alpha \;\frac{e_{31,f}}{\varepsilon_{33,f}}$$

Mass loading of a piezoelectric vibrating membrane in the case of a gas sensing mechanism involves adsorption of the target gas molecules on the sensing layer on top of the thin film electrode. This mechanism of a sensor is altered and determined by two important parameters, resonant frequency and sensitivity. The shift in the resonance frequency is due to changes in acoustic impedance mismatching and change in mass of the overall structure. The interaction between the analytes and the sensor is the cause of the change in the mass either due to absorption or adsorption. The following equation shows the general relation between the mass loading and the change in frequency [44].

(24) $$∆f_{m}=-K\;\cdot \;S_{m\;}\cdot \;∆m_{A}$$

where $S_{m\;}$ is the nature of the piezoelectric substrate, the dimensions of the structure, the resonant frequency and the acoustic mode, *K* is geometric factor of active area and $∆m_{A}$ is change in mass over area. Based on the application, the resonant frequency is determined by the material properties and the dimensions in vibrating membranes. For a rectangular PMUT structure, the resonant frequency *f_0_* is given by Equation (25) [46]

(25) $$f_{0}=\frac{0.494t}{w^{2}}\sqrt{\frac{E}{\rho \left(1-\upsilon^{2}\right)}\left[1+\frac{2}{3}{\left(\frac{W}{L}\right)}^{2}+{\left(\frac{W}{L}\right)}^{4}\right]}$$

where *t* is the thickness, *L* is the length, *w* is the width, *E* is Young’s modulus, ρ is Poisson’s ratio, and υ is the density of the material. From the above equation, the shape, the radius and the thickness of both the electrodes and piezo-material alter the resonant frequency, which in turn affects the sensitivity. The density and material property of the sensing layer plays a predominant role in determining the overall performance of the PMUT sensor. Electromechanical coupling, *K^2^_eff_,* provides the conversion between electrical energy and mechanical energy which is shown in Equation (26).

(26) $$K_{eff}^{2}=1-{\left(\frac{f_{r}}{f_{a}}\right)}^{2}$$

where *f_r_* is the resonant frequency and *f_a_* is the anti-resonant frequency, which is determined by the piezoelectric layer’s thickness.

Sensitivity determines the performance of a sensor. The ratio of change in frequency to change in mass added on the sensing layer is the sensitivity of the sensor. Here, the mass refers to the surrounding gas. Altering a few physical properties such as mass over area, Young’s modulus, viscoelasticity, or electrical conductivity of the thin films impacts the sensitivity. A PMUT sensor works based on frequency shift, when exposed to target gas molecules, which is dependent on the fundamental resonant frequency (*f_o_*), mass of the vibration membrane and the change in mass after the adsorption of the target gas molecules. The frequency shift is estimated by

(27) $$\Delta f=-\frac{1}{2}f_{0}\times \frac{\Delta m}{m}$$

where *f_o_* is the fundamental resonant frequency, *m* is the mass of the vibrating membrane and *Δm* is the mass change after the absorption of target gas molecules. For sensing applications, thin film based PMUT devices are shown to be promising candidates with piezoelectric membrane such as aluminum nitride (AlN). AlN has low permittivity and its residual stress can be avoided as AlN does not require an annealing process [46]. 

**Figure 10:**
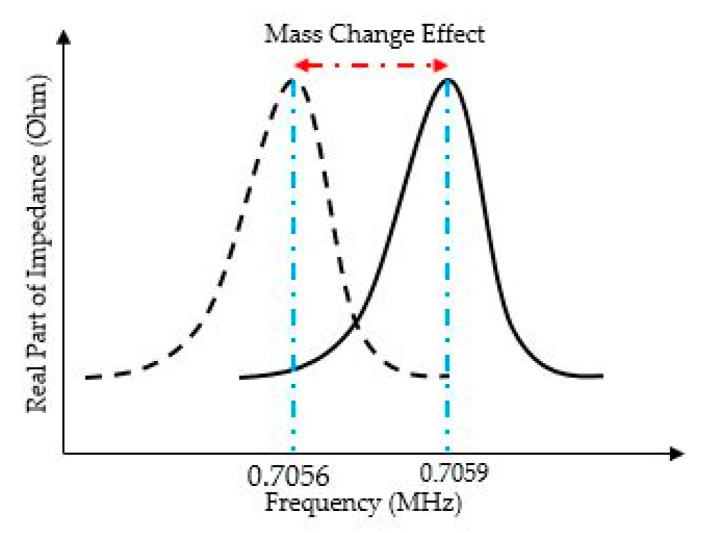
Frequency shift in response to the mass change.

As shown in Figure 10, a frequency shift of 300 Hz is achieved for a simulated PMUT-based sensor. This device is functionalized by a 500 nm PIB layer when the radius is 250 μm and the membrane thickness is 500 nm. A 10 V AC signal is applied to the top electrode. The device provides a sensitivity of 66.4 Hz/ng. 

Scaling down the size of the PMUT membrane results in a reduction in not only the absolute value of the effective mass of the vibrating membrane but also its dimensions, geometry and resonance mode. There exist challenges such as fluctuations in the temperature, pressure changes, mechanical fracture, electrical short circuits and open circuit, and oxidation of the sensing element. Passive layers such as SiO_2_ and Si, location of the top electrode, shape of the device and the piezoelectric constant directly control the performance of a piezoelectric thin film PMUT sensor [47]. A PMUT sensor can be functionalized using graphene, or any polymer where the change in mass before and after adsorption of water molecules showcases PMUT as a highly sensitive sensor to humidity [46].

### 3.3. Fabrication Techniques for PMUT

The predominantly used techniques to fabricate PMUTs are discussed here. In general, silicon (Si) or silicon dioxide (SiO_2_) are used as the substrate. The alteration of the piezoelectric layer in the PMUT structure involves either metal deposition by sol-gel or sputtering. The cavity formation has several options, such as sacrificial layer releasing and back side etching.

#### Sacrificial Technique

**Figure 11:**
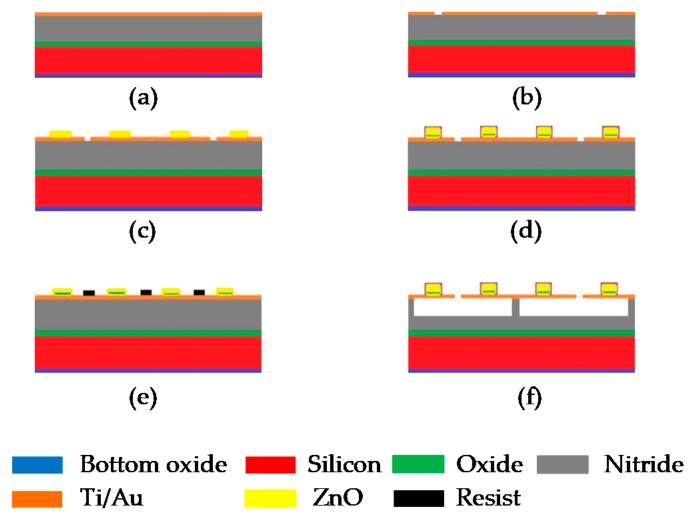
Fabrication of PMUT using the sacrificial method. (**a**) The silicon layer is doped by low-pressure chemical vapor deposition (LPCVD) method and the bottom electrode is deposited by e-beam evaporation, (**b**) the access holes are made on the bottom electrode layer by wet etching and then patterned, (**c**) the deposition of piezo layer by the sputtering method, (**d**) the deposition of top electrode using the e-beam evaporation and patterned using the process of lift-off and (**e**) the resist is placed to protect the access holes, (**f**) the wet etching is done on sacrificial layer.3.3.2. Back Side Etching.

The sacrificial layer method is one of the commonly used PMUT fabrication techniques. In this method, a sacrificial layer is used to define the structure of the PMUT. E-beam evaporation is used to deposit the bottom electrode layer, as shown in Figure 11a, on a doped silicon substrate. The access holes are made on the bottom electrode layer by wet etching and then patterned, as illustrated in Figure 11b. After this step, the piezo-layer is sputtered followed by the deposition of the top electrode using the E-beam evaporation, and then it is patterned using the process of lift-off. Conclusively, the wet etching is done on the sacrificial layer, as shown in Figure 11c–f. This technique has been reported to be used for membranes with radii ranging from tens to hundreds of microns [48].

**Figure 12:**
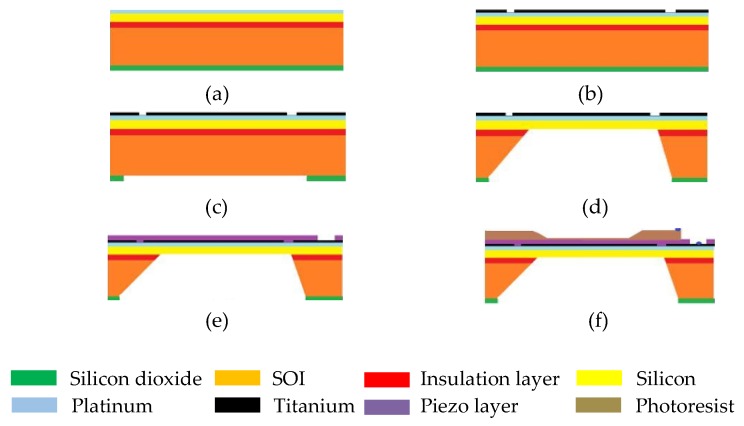
The fabrication of PMUT using the back-etching method. (**a**) The silicon is oxidized, insulation layer is created for bottom electrode and mask layer is created for the top electrode, (**b**) the top and bottom electrodes are deposited by sputtering and patterned, (**c**) the silicon dioxide layer is patterned, (**d**) the silicon is etched till insulation layer to create the diaphragm, (**e**) the deposition of piezoelectric layer either by sputtering or Sol-gel method, (**f**) the photoresist is formed and patterned.

Another common technique to fabricate PMUT is the back-side etching method. The thinning of the silicon wafer and the back-end process is done by the wet etching process. This process is low cost and reliable with a high throughput. Based on the etch stop and etch rate, the frequently used wet etchants are potassium hydroxide (KOH) and tetramethylammonium hydroxide (TMAH). However, the backside etching process degrades or damages the silicon layer and creates a residual stress which reduces the size of the pitch and the diaphragm. In this process and in order to create an insulation and a mask layer for bottom and top electrodes, the substrate is oxidized, as illustrated in Figure 12a. The thin film depositions of the top and bottom electrodes are done by sputtering, and the layers are patterned to create the electrodes as shown in Figure 12b. The piezoelectric layer is prepared by the sol-gel method, as shown in Figure 12c. The oxidized layer is then patterned to create the diaphragm pattern followed by anisotropic etching as shown in Figure 12d. The etching is done up to the SOI wafer and then the insulation layer, formed at the beginning, is removed, as illustrated in Figure 12e,f. The backside etching technique has been reported to be used for backside cavities in PMUT-based sensor fabrication ranging over hundreds of microns [49,50].

## 4. Summary 

In an unconventional approach, CMUTs and PMUTs can be configured as gas sensors, operating based on the change in mass of their sensing layer. CMUT gas sensors consist of a suspended membrane on top of a fixed bottom electrode to create a dynamic capacitance when the DC bias voltage is applied to the membrane, whereas the PMUTs employed in gas sensing technology benefit from the properties of piezoelectric materials when the membrane deflects and the DC bias voltage is applied to the piezo-material. CMUT gas sensors present a lower limit of detection and a high mass sensitivity of Hz/zg alongside a higher bandwidth than PMUT, while the reported sensitivity for PMUT is Hz/% relative humidity. Furthermore, thin film PMUTs exhibit unique properties because of their lower required operating voltage. Moreover, PMUT sensors used in a linear array with a sensing polymer can detect volumetric changes in the nanoscales. CMUT structures usually operate with higher voltage and can provide high frequencies in comparison to the PMUT configuration. Conclusively, low energy consumption, potential for high sensitivity, and possibility of array fabrication make CMUT- and PMUT-based mass sensors potential candidates for sensing in complex environments.
